# Anterior Cingulate Cortex Astrocytes Regulate Chronic Itch and Comorbid Negative Emotional Disorders in Mice

**DOI:** 10.1002/cns.71165

**Published:** 2026-09-18

**Authors:** Ze Fan, Xiaotong Shi, You Wu, Ningcan Ma, Ziyi Dai, Lisen Wang, Yushan Jin, Yuanyuan Zhu, Yiming Chen, Haopeng Zhang, Hui Zhang, Shengxi Wu, Jing Huang

**Affiliations:** ^1^ Department of Neurobiology, School of Basic Medicine The Fourth Military Medical University Xi'an China; ^2^ The Shaanxi Province Key Laboratory of Brain Function Analysis and Modulation Xi'an China; ^3^ State Key Laboratory of Oral & Maxillofacial Reconstruction and Regeneration, National Clinical Research Center for Oral Diseases, Shaanxi Engineering Research Center for Dental Materials and Advanced Manufacture, Department of Anesthesiology, School of Stomatology The Fourth Military Medical University Xi'an China; ^4^ The Key Laboratory of Neural and Vascular Biology, Ministry of Education Hebei Medical University Shijiazhuang China

**Keywords:** anterior cingulate cortex, anxiety, astrocytes, chronic itch, depression

## Abstract

**Aims:**

The anterior cingulate cortex (ACC) is widely recognized as a key brain region in modulating itch and itch‐associated behaviors. Though neuronal mechanisms are increasingly clarified, the role of ACC astrocytes remains poorly understood.

**Methods:**

By employing a diphenylcyclopropenone (DCP)‐induced chronic itch mouse model, we assessed scratching behavior and negative emotions via behavioral tests. Astrocytic morphology was examined through immunofluorescence staining and sholl analysis. Fiber photometry was used to record calcium signals of ACC astrocytes and CaMKII‐positive neurons. Chemogenetic manipulation was applied to modulate astrocyte activity. Gliotransmitter quantification was implemented to detect the gliotransmitter release in the ACC.

**Results:**

DCP induced robust scratching responses and concomitant anxiety‐ and depression‐like behaviors. ACC astrocytes exhibited morphological activation and elevated calcium responses. Chemogenetic activation of ACC astrocytes alleviated DCP‐induced scratching behavior and negative emotions, and enhanced calcium activity of local CaMKII‐positive neurons. Conversely, chemogenetic inhibition of ACC astrocytes reduced such neuronal calcium activity. D‐serine and ATP levels were reduced in DCP‐induced chronic itch, while chemogenetic activation of astrocytes significantly elevated them.

**Conclusions:**

ACC astrocytes play a critical role in regulating chronic itch and comorbid anxiety‐depression by modulating CaMKII‐positive neuronal activity, providing a potential therapeutic target for intractable chronic itch in clinical settings.

## Graphical abstract

ACC astrocytes are morphologically and functionally activated in DCP‐induced chronic itch. Chemogenetic activation of these astrocytes positively modulates the calcium activity of local CaMKII^+^ excitatory neurons and the release of D‐serine and ATP, and exerts anti‐pruritic, anxiolytic, and anti‐depressant effects.

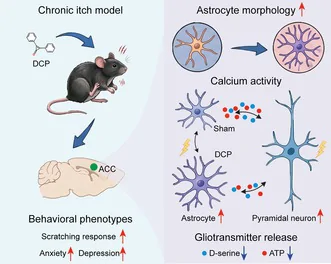

## 1 Introduction

Itch, or pruritus, is a discomforting perceptual experience that elicits the urge to scratch [1]. It represents a common but debilitating clinical symptom across dermatological, renal, and hepatobiliary diseases [2, 3, 4]. Unlike acute itch, which serves as an evolutionarily defensive mechanism against noxious external stimuli, chronic itch persists for weeks to years and is frequently comorbid with anxiety and depression, exerting a devastating impact on patients' quality of life [5, 6]. Although peripheral pruritic mediators and central neural circuits underlying itch processing have been progressively uncovered [7, 8], the cortical mechanisms governing pruritic signal processing and its associated emotional comorbidities remain largely elusive [9, 10].

The anterior cingulate cortex (ACC), a core limbic region, is well recognized for its pivotal role in encoding the affective and motivational dimensions of sensory stimuli, including pain and itch [11, 12]. Functional neuroimaging studies in humans have revealed that abnormal ACC hyperactivity is closely correlated with the severity of chronic itch and its comorbid anxiety and depression [13]. Consistently, preclinical studies have demonstrated that chemogenetic suppression of ACC neurons markedly attenuates itch and negative emotions [14]. Specific neuronal subpopulations within the ACC, including CaMKII‐positive excitatory neurons and distinct subsets of GABAergic interneurons, have been identified to exert differential modulatory effects on itch processing [15]. Despite substantial progress in investigating neuronal mechanisms of the ACC, the role of glial components within the ACC in modulating itch sensation and associated negative emotions remains largely unknown, hindering a comprehensive understanding of the complex cellular networks governing chronic itch and its comorbid negative emotional symptoms.

Astrocytes are the most abundant and functionally complex glial cells in the central nervous system [16]. Through their highly differentiated elaborate morphology and extensive intercellular interactions, astrocytes form tight structural and functional integration with neuronal synapses, exerting a pivotal regulatory role in maintaining synaptic homeostasis and plasticity, as well as the overall functional performance of neural networks [17, 18]. In the spinal cord, astrocytic activation has been suggested to amplify itch signaling via the release of pro‐inflammatory cytokines and gliotransmitters [19, 20]. In the rostral ventromedial medulla (RVM), astrocyte reactivity is a key contributor to chronic itch pathogenesis and its associated anxiety‐like behaviors [21]. Whether and how ACC astrocytes respond to chronic itch, and their role in modulating local neuronal activity as well as itch‐emotion comorbidity, remain poorly understood.

Herein, we employed a diphenylcyclopropenone (DCP)‐induced chronic itch mouse model, combined with immunofluorescence staining, sholl analysis, fiber photometry, chemogenetic manipulation, and gliotransmitter quantification, to systematically investigate the function of ACC astrocytes in chronic itch and comorbid emotional disorders. We found that DCP induced morphological and functional activation of ACC astrocytes. Selective chemogenetic activation of ACC astrocytes ameliorated DCP‐induced scratching behavior and comorbid anxiety‐ and depression‐like behaviors. Additionally, ACC astrocytes positively modulated local CaMKII‐positive neuronal activity. Quantification of gliotransmitters revealed that DCP elicited a significant reduction in D‐serine and ATP levels in the ACC, and chemogenetic activation of astrocytes reversed these deficits. This study elucidates the cortical astrocyte‐neuron interaction mechanism underlying itch processing and associated negative emotions, offering a promising therapeutic target for clinical management.

## 2 Materials and Methods

### 2.1 Animals

Adult male C57BL/6J mice were obtained from the Experimental Animal Center of the Fourth Military Medical University. Aldh1l1‐Cre^ERT2^ mice were generated from the Jackson Laboratory (MGI ID: 5806568). All mice were maintained in a standardized animal housing facility with controlled environment: temperature at 22°C–25°C, humidity at 50%, and a 12‐h light/dark cycle, with ad libitum access to standard laboratory chow and drinking water. All animal procedures were approved by the Institutional Animal Care and Use Committee at the Fourth Military Medical University (NO: IACUC‐20250181) and complied with the National Institutes of Health (NIH) Guide for the Care and Use of Laboratory Animals.

### 2.2 Chronic Itch Model

DCP (177,377, Sigma‐Aldrich, USA) was utilized to establish a contact dermatitis model as previously described [22]. Briefly, mice were anesthetized with 2% isoflurane (R510‐22‐10, RWD Life Science Inc., China), and the nape hair was shaved to expose target skin for subsequent treatment. For sensitization, 200 μL DCP solution (1% w/v in acetone) was applied to the depilated nape skin. After a 1‐week interval, mice underwent daily administration of 0.5% DCP solution for seven successive days. Sham mice received identical acetone vehicle treatment. Scratching responses were video‐recorded for 30 min and quantified by a blinded investigator.

### 2.3 Behavioral Tests

Mice were randomly assigned to experimental groups and acclimated to the testing room at least 1 h before testing. All apparatuses were cleaned with 75% ethanol to minimize residual odors and excreta, and all behavioral analyses were conducted in a blinded manner.

#### 2.3.1 Scratching Bouts

Each mouse was individually positioned in a clear glass‐bottom plastic enclosure for sufficient habituation, followed by 30 min of continuous video recording. A scratching bout was defined as hind paw rubbing on the target neck skin, and total bouts were manually counted.

#### 2.3.2 Open Field Test (OFT)

Mice were positioned in the central area of an open field apparatus (50 × 50 × 40 cm) and allowed to explore freely for 10 min under a video‐recording system. Total distances, time in center zone, and central zone entries were analyzed with SMART software (version 3.0, Panlab, Spain) to evaluate locomotor activity and anxiety‐like behavior.

#### 2.3.3 Elevated Plus Maze (EPM)

Mice were positioned onto the central platform of the EPM apparatus (two open arms: 30 × 5 cm; two closed arms: 30 × 5 cm with 30 cm‐high walls; 50 cm above ground), and permitted to explore freely for 5 min under a video‐recording system. Similarly, total distance, time in open arm, and open arm entries were analyzed with SMART software (version 3.0) to assess anxiety‐like phenotypes.

#### 2.3.4 Tail Suspension Test (TST)

Mice were suspended 50 cm above the ground by the tail tip for 6 min, with the first 2 min set as habituation. Immobility time during the final 4 min was quantified to reflect behavioral despair.

#### 2.3.5 Forced Swimming Test (FST)

Mice were placed into an open cylindrical tank (diameter 18 cm, height 30 cm) filled with water maintained at 24°C ± 1°C to a depth of 15 cm. Each mouse underwent a 6‐min swimming session recorded by video camera. The first 2 min served as habituation, and immobility time during the subsequent 4 min was video‐recorded and blindly analyzed to evaluate behavioral despair. Mice were gently dried with a towel after each test before being returned to their home cages.

#### 2.3.6 Sucrose Preference Test (SPT)

Mice were single‐housed and acclimated to the testing environment for 1 h before the experiment. The test consisted of four sequential phases: a 24 h adaptation phase with two water bottles exchanged every 6 h; a 24 h training phase with one bottle of 1% sucrose solution and one bottle of water, with bottle positions swapped every 6 h; a 24 h deprivation phase without food and water; and a 12 h formal testing phase with simultaneous access to sucrose solution and water. Bottle weights were recorded before and after testing. Sucrose preference was calculated as sucrose consumption/(sucrose consumption + water consumption) × 100%.

#### 2.3.7 Novelty‐Suppressed Feeding Test (NSFT)

Following 24 h food deprivation, mice were placed in a brightly lit open‐field arena with a food pellet positioned at the center. The latency to contact or eat the food within 10 min was recorded. Home‐cage food consumption within 5 min was further measured to exclude general appetite changes. All behavioral analyses were performed blindly.

### 2.4 Immunofluorescence Staining

Mice were anesthetized with 2% pentobarbital sodium (50 mg/kg, intraperitoneal injection), then perfused with precooled phosphate‐buffered saline (PBS, 0.01 M) and 4% paraformaldehyde (PFA). Brains were dissected and post‐fixed in 4% PFA, and then dehydrated in gradient sucrose solutions. Coronal sections (30 μm thick) were cut using a cryostat (CM1905, Leica, Germany). Free‐floating sections were incubated in blocking buffer consisting of 3% bovine serum albumin (BSA, A1933, Sigma) and 0.3% Triton X‐100 (T9284, Sigma) for 1 h, then incubated overnight at 4°C with primary antibodies as listed in Table S1. After thorough rinsing in PBS, sections were incubated with species‐matched fluorescent secondary antibodies for 2 h at room temperature. Nuclei were counterstained using DAPI (1:1000, D9564, Sigma), and fluorescent images were captured using a confocal laser scanning microscope (Olympus Fluoview Ver4.2b, Japan).

### 2.5 3D Reconstruction of Astrocytes

rAAV‐GfABC1D‐EGFP (200 nL, BC‐0377, Brain Case Ltd., China) was bilaterally microinjected into the ACC as previously described [23]. Three weeks after virus expression, brain slices were collected and Z‐stack images were captured via a laser scanning confocal microscope fitted with a 100× objective lens, with a z‐step interval of 0.5 μm. Imaris software (version 9.3.1, Bitplane, Switzerland) was used for background subtraction, Gaussian filtering, and 3D reconstruction. Astrocyte morphological parameters, including process length, branch number, surface area, and process intersections, were automatically quantified for morphological assessment.

### 2.6 Virus Injection

Mice were anesthetized with 2% isoflurane and placed in a stereotaxic head holder (68,018, RWD Life Science Inc.). Intracranial virus injection targeting the ACC was performed at stereotaxic coordinates as follows: anterior–posterior: +0.80 mm, medial‐lateral: ±0.30 mm, dorsal‐ventral: −1.75 mm. A total volume of 200 nL virus was injected at a rate of 30 nL/min using a Hamilton syringe (CN‐07940‐07, Switzerland) connected to a microprocessor‐controlled injector (Legato 210, KD Scientific, USA). The injection needle was retained for 10 min post‐injection to prevent backflow and ensure sufficient viral diffusion. Mice were placed in a warm chamber for full recovery after surgery.

### 2.7 Fiber Photometry

For calcium recording, rAAV‐GfABC1D‐cyto‐GCaMP6f (BC‐0378, Brain Case Ltd., China) or rAAV‐CaMKII‐GCaMP6s (BC‐0081, Brain Case Ltd.) was injected into the ACC to label astrocytes and excitatory neurons, respectively. After DCP modeling and 2‐week recovery, optical fibers were implanted for signal recording. A fiber photometry system (FPS‐410/470, Inper Ltd., China) containing 470 nm excitation laser and 410 nm isosbestic reference laser were used to capture calcium signals during scratching behaviors (Figure 2K). Animals with robust viral expression, accurate stereotaxic fiber placement and stable behavioral performance were included, with at least five biological replicates per group according to published literature [15, 24, 25, 26]. ΔF/F was calculated as (F − F0)/F0, with F0 defined as baseline fluorescence and F denoting the instantaneous fluorescence intensity at any individual time point. The baseline (−2–0 s) and scratching onset (0–2 s) periods were defined, and signal area under curve (AUC) was quantified to evaluate calcium activity changes via Inper Plot software (v.1.9.6, China).

### 2.8 Chemogenetic Manipulation

Aldh1l1‐Cre^ERT2^ mice received daily tamoxifen intraperitoneal injection (100 mg/kg, T5648, Sigma) for seven successive days to induce Cre recombination [27]. Subsequently, bilateral stereotaxic injections of 200 nL Cre‐dependent viral vectors were performed targeting the ACC as follows: rAAV‐EF1α‐DIO‐hM3D(Gq)‐mCherry (BC‐0146, Brain Case Ltd.), rAAV‐EF1α‐DIO‐hM4D(Gi)‐mCherry (BC‐0155, Brain Case Ltd.), and rAAV‐EF1α‐DIO‐mCherry (BC‐0016, Brain Case Ltd.). The DCP‐induced chronic itch model was established 1 week after virus injection. Two weeks post‐modeling, mice were intraperitoneally administered clozapine N oxide (CNO, 0.75 mg/kg, 4936, Tocris, UK) 30 min before behavioral tests to modulate astrocyte activity (Figure 4A).

### 2.9 Brain Slice Ca^2+^ Imaging

To investigate the dynamic fluctuations of calcium signals in the soma, large branches, and territories of astrocytes, rAAV‐GfABC1D‐cyto‐GCaMP6f was stereotaxically microinfused into the mouse ACC. Three weeks later, 200 μm acute ACC slices were prepared in ice‐cold oxygenated calcium‐free artificial cerebrospinal fluid (ACSF) with a vibratome (VT‐1200S, Leica, Germany) at a cutting speed of 0.1 mm/s. Then, slices were recovered in 34°C oxygenated ACSF for 30 min before imaging to ensure the reliability of cell function and calcium signal detection. Continuous imaging was performed for 300 s at 1 s frame intervals using a confocal microscope (Version 4.2b, Olympus Fluoview). Adenosine triphosphate (ATP, 100 μM) was added at the 20‐s time point to induce calcium responses. CellSense software (version 4.4.1, Olympus) was used to analyze calcium peak amplitude and AUC in three astrocyte sub‐regions: soma, large branches, and fine territories.

### 2.10 Gliotransmitter Quantitative Detection Kits

ACC tissues (10 mg/sample) were prepared and homogenized on ice, followed by quantitative determination of various gliotransmitters via corresponding commercial detection kits as shown in Table S2. All steps were strictly performed in compliance with the detailed operating instructions provided by the kit manufacturers to guarantee the precision and repeatability of quantitative measurements.

### 2.11 Statistical Analysis

Statistical analyses were conducted with GraphPad Prism software (version 9.0). All data were presented as mean ± standard deviation (SD). Data normality and variance homogeneity were examined using the Shapiro Wilk and Levene's tests, respectively. For data that satisfied both the normality and variance homogeneity criteria, statistical comparisons were performed using either a two‐tailed unpaired *t*‐test or two‐way ANOVA followed by Bonferroni's post hoc test. In cases where data did not meet these criteria, nonparametric statistical methods were adopted, including the Mann–Whitney *U* test or Welch's *t*‐test (Details in Table S3). Differences were considered statistically significant at *p* < 0.05.

## 3 Results

### 3.1 DCP Induces Increased Scratching Bouts and Comorbid Anxiety‐ and Depression‐Like Behaviors in Mice

**FIGURE 1:**
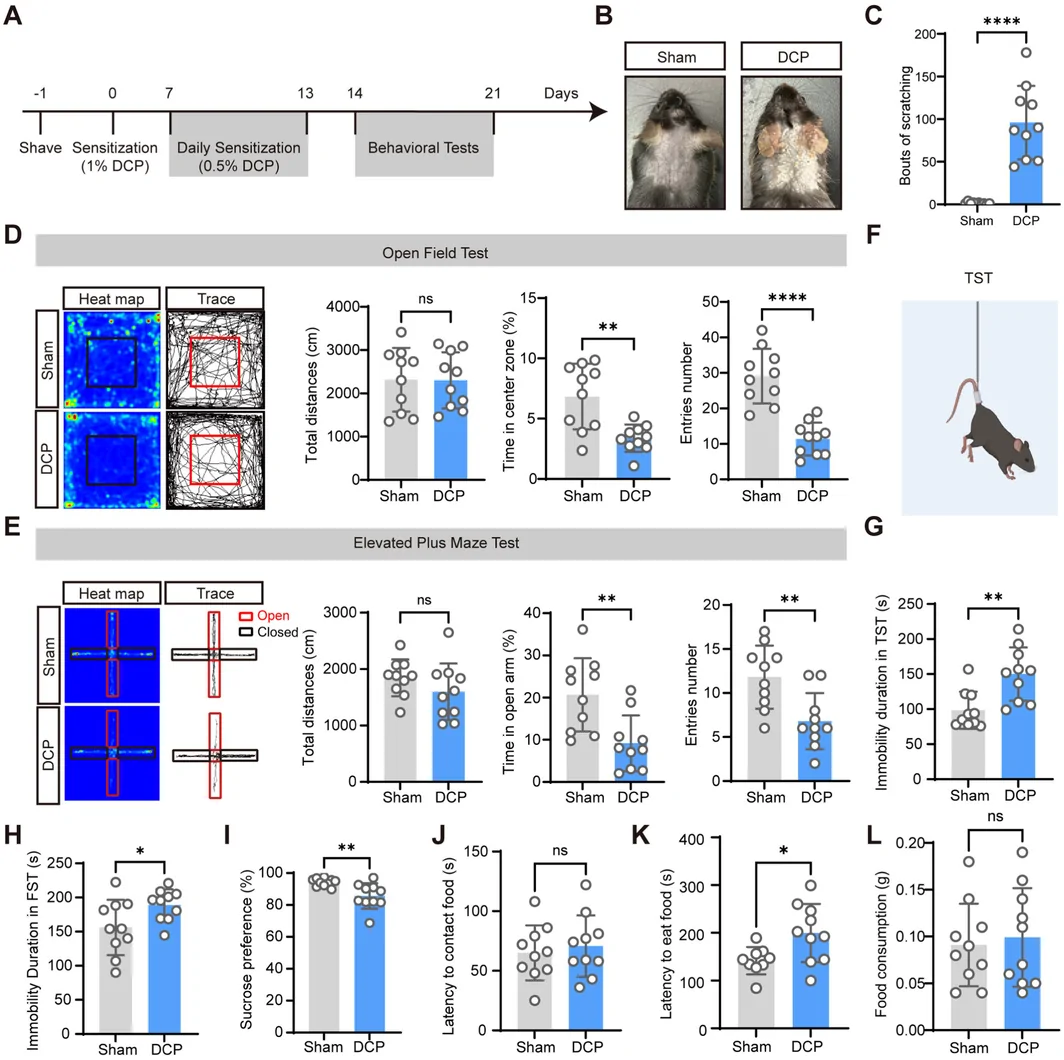
DCP induces chronic itch and comorbid anxiety‐ and depression‐like behaviors in mice. (A) Schematic diagram of DCP‐induced chronic itch model and behavioral tests. (B) Representative images of skin lesion in Sham and DCP‐treated mice. (C) Number of scratching bouts in Sham and DCP groups. (D) Representative heatmaps and movement trajectories, and quantification of total distance traveled, time spent in center zone, and number of center zone entries in the open field test. (E) Representative heatmaps and movement trajectories, and quantification of total distance traveled, time spent in open arm, and number of open arm entries in the elevated plus maze. (F) Schematic diagram of tail suspension test. Quantification of (G) immobility time in the TST, (H) immobility time in the FST, (I) sucrose preference in the SPT, (J) latency to contact food, (K) latency to eat food, and (L) food consumption in the NSFT. *n* = 10 mice per group. Data are presented as mean ± SD, *p < 0.05, **p < 0.01, ****p < 0.0001.

A 2‐week DCP‐induced sensitization protocol was used to establish chronic itch model (Figure 1A). Macroscopic examination of the nape skin revealed that DCP‐treated mice developed prominent skin erythema, scaling, and excoriation (Figure 1B), accompanied by significantly increased scratching bouts compared with Sham group (Figure 1C), confirming the successful model construction. To evaluate affective comorbidities, anxiety‐like behaviors were examined via OFT and EPM. In the OFT, no significant difference in total locomotor distance was detected between groups, ruling out confounding effects on general motor activity. However, DCP‐treated mice displayed a marked reduction in both the percentage of time spent in the central zone and the number of central zone entries (Figure 1D), indicative of heightened anxiety‐like behavior. Similarly, in the EPM, DCP‐treated mice displayed fewer open arm entries and less time spent in open arms, with no significant change in total distance (Figure 1E). Depression‐like behavior was quantified using the TST, FST, SPT and NSFT. We found that DCP‐treated mice exhibited a significant increase in immobility time during the testing period in TST and SPT (Figure 1F–H), reflecting enhanced behavioral despair. Besides, the sucrose preference in SPT was declined (Figure 1I), and latency to eat food in NSFT was increased (Figure 1K), reflecting anhedonia and motivational deficits in DCP‐treated mice, though the latency to contact food and home‐cage food consumption were not significantly changed in NSFT (Figure 1J,L). Collectively, these results demonstrate that DCP induces robust chronic itch and comorbid anxiety‐ and depression‐like behaviors.

### 3.2 Astrocytic Responses in the ACC During DCP‐Induced Chronic Itch Processing

**FIGURE 2:**
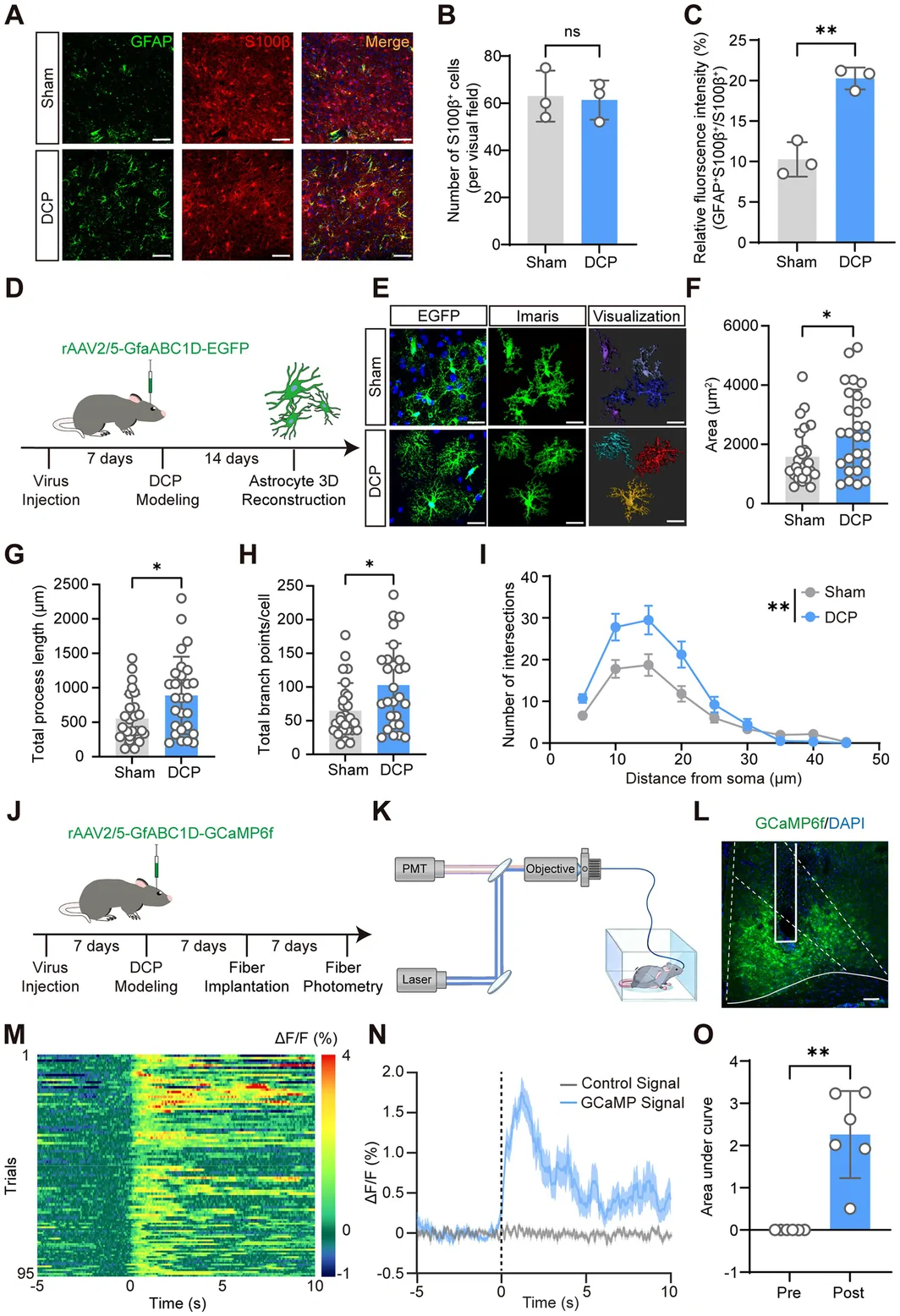
Morphological analysis of ACC astrocytes and calcium activity during scratching behavior. (A) Representative images of immunofluorescence staining of GFAP and S100β in the ACC of Sham and DCP group mice. Scale bar = 50 μm. Statistical analysis of (B) S100β‐positive cells and (C) proportion of GFAP‐positive cells in GFAP/S100β‐positive cells in the ACC. *n* = 3 per group. (D) Schematic diagram of viral strategy and 3D reconstruction of astrocytes. (E) Representative images, (F) quantification of the area, (G) total processes length, (H) number of branch points, and (I) sholl analysis of intersections of astrocytes in the ACC. Scale bar = 20 μm. *n* = 27 cells from 3 mice per group. (J) Schematic diagram of fiber photometry recording. (K) The fiber photometry setup. (L) Histological verification of viral expression and optical fiber implantation in the ACC. Scale bar = 100 μm. (M) Heatmap of GCaMP6f fluorescence aligned to scratching bout onset across all mice. (N) Mean fluorescent response to scratching behavior. (O) Area under the curve showing fluorescence changes during pre‐ and post‐scratching periods. *n* = 6 mice per group. Data are presented as mean ± SD, *p < 0.05, **p < 0.01.

**FIGURE 3:**
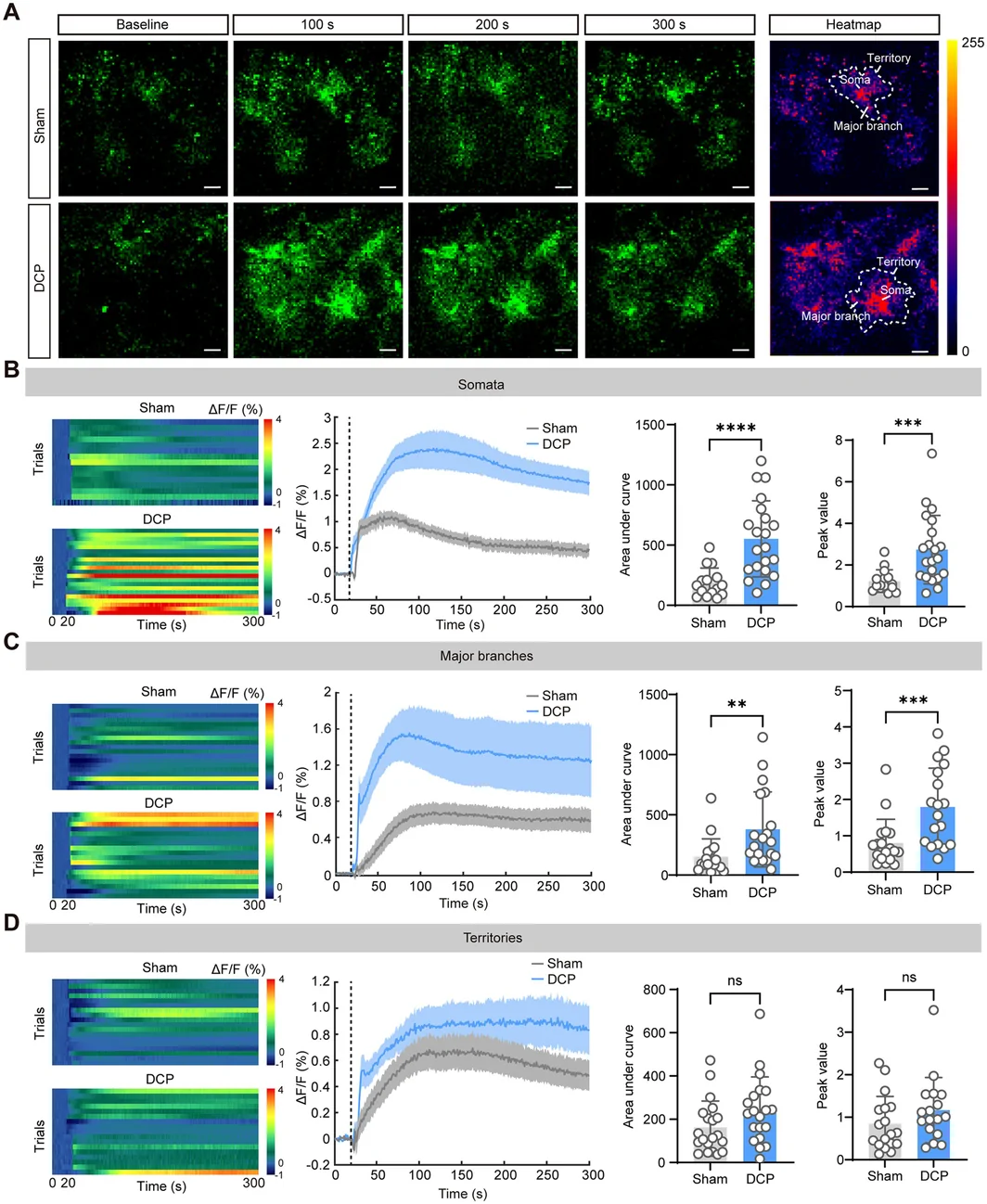
Calcium responses in somata, major branches, and terminal branches of ACC astrocytes in DCP‐treated mice. (A) Representative images and heatmap of ex vivo calcium imaging in ACC brain slices. Scale bar = 20 μm. (B–D) Heatmap, mean fluorescent response and statistical analysis of area under the curve and peak amplitude of calcium transients in somata (B), major branches (C), and terminal branches (D) of astrocytes in Sham and DCP group mice. *n* = 15–21 cells from 3 mice per group. Data are presented as mean ± SD, **p < 0.01, ***p < 0.001, ****p < 0.0001.

Considering the pivotal role of ACC in integrating sensory and affective components in pruritus and nociception, we explored the morphological and functional plasticity of ACC astrocytes in DCP‐induced chronic itch. Immunofluorescence staining revealed no significant alteration in the number of S100β^+^ astrocytes between groups. However, GFAP fluorescence intensity was markedly increased in DCP group (Figure 2A–C). Subsequently, we performed 3D reconstruction of ACC astrocytes for a more detailed morphological assessment (Figure 2D,E). Morphometric analysis demonstrated that ACC astrocytes in the DCP‐treated mice exhibited enlarged surface area, increased total process length, elevated branch number, and a higher density of process intersections (Figure 2F–I). To assess functional activity, we utilized fiber photometry to record calcium transients during spontaneous scratching bouts (Figure 2J–L). We found that DCP‐treated mice displayed a robust enhancement in astrocytic calcium signal AUC during scratching episodes (Figure 2M–O). Ex vivo brain slice imaging further dissected the compartment‐specific calcium activation. We found that ATP‐evoked calcium peak amplitude and AUC were significantly amplified in the soma and large branches of ACC astrocytes in DCP‐treated mice, while fine terminal territories remained unchanged (Figure 3A–D). Collectively, these findings demonstrate that DCP‐induced chronic itch elicits subregion‐specific morphological and functional activation of ACC astrocytes.

### 3.3 Chemogenetic Activation of Astrocytes in the ACC Alleviates Chronic Itch and Comorbid Emotional Disorders

**FIGURE 4:**
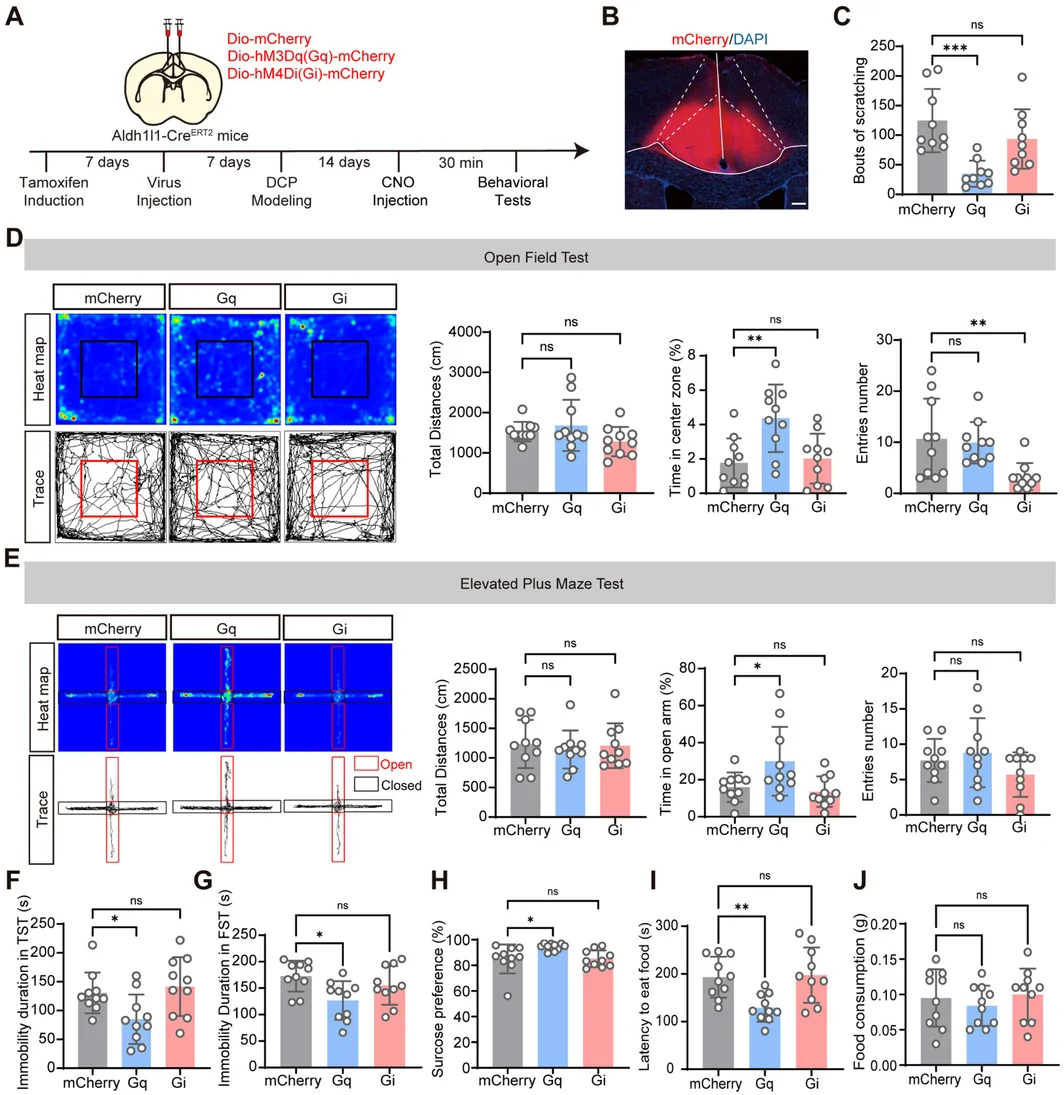
Chemogenetic activation of ACC astrocytes alleviates DCP‐induced chronic itch and negative emotions. (A) Schematic diagram of chemogenetic manipulation of ACC astrocytes in Aldh1l1‐Cre^ERT2^ mice. (B) Representative images confirming mCherry expression in the ACC. Scale bar = 200 μm. (C) Chemogenetic activation of ACC astrocytes reduces scratching behaviors induced by DCP, whereas chemogenetic inhibition exerts no effects. (D, E) Representative locomotion trajectories, heatmaps and quantification of mice in the mCherry, Gq, and Gi groups in the open field test (D) and elevated plus maze (E). Quantification of (F) immobility time in the TST, (G) immobility time in the FST, (H) sucrose preference in the SPT, (I) latency to eat food and (J) food consumption in the NSFT in the mCherry, Gq, and Gi group mice. *n* = 10 mice per group. Data are presented as mean ± SD, *p < 0.05, **p < 0.01, ***p < 0.001.

We utilized Aldh1l1‐Cre^ERT2^ mice and cre‐dependent chemogenetic viruses to bidirectionally modulate ACC astrocyte activity (Figure 4A,B). Behavioral results demonstrated that chemogenetic activation of ACC astrocytes (Gq group) significantly reduced DCP‐induced scratching bouts (Figure 4C). Concomitantly, anxiety‐like behaviors were markedly ameliorated, with a notable increase in both the percentage of time spent in the central zone and open arms in the OFT and EPM, respectively (Figure 4D,E). Furthermore, chemogenetic activation of ACC astrocytes relieved depression‐like behavior, characterized by a pronounced decrease in immobility time during the TST and FST (Figure 4F,G), a significant increase in SPT (Figure 4H), as well as a reduced latency to eat food in NSFT (Figure 4I), though the home‐cage food consumption was not significantly changed (Figure 4J). Chemogenetic inhibition of ACC astrocytes (Gi group) had no significant impact on DCP‐induced chronic itch or its comorbid affective phenotypes (Figure 4C–J). Collectively, these findings indicate that enhanced ACC astrocyte activity exerts anti‐pruritic and anxiolytic‐depressant effects in chronic itch.

### 3.4 ACC Astrocytes Positively Modulate CaMKII ^+^ Neuronal Activity in DCP‐Induced Chronic Itch

**FIGURE 5:**
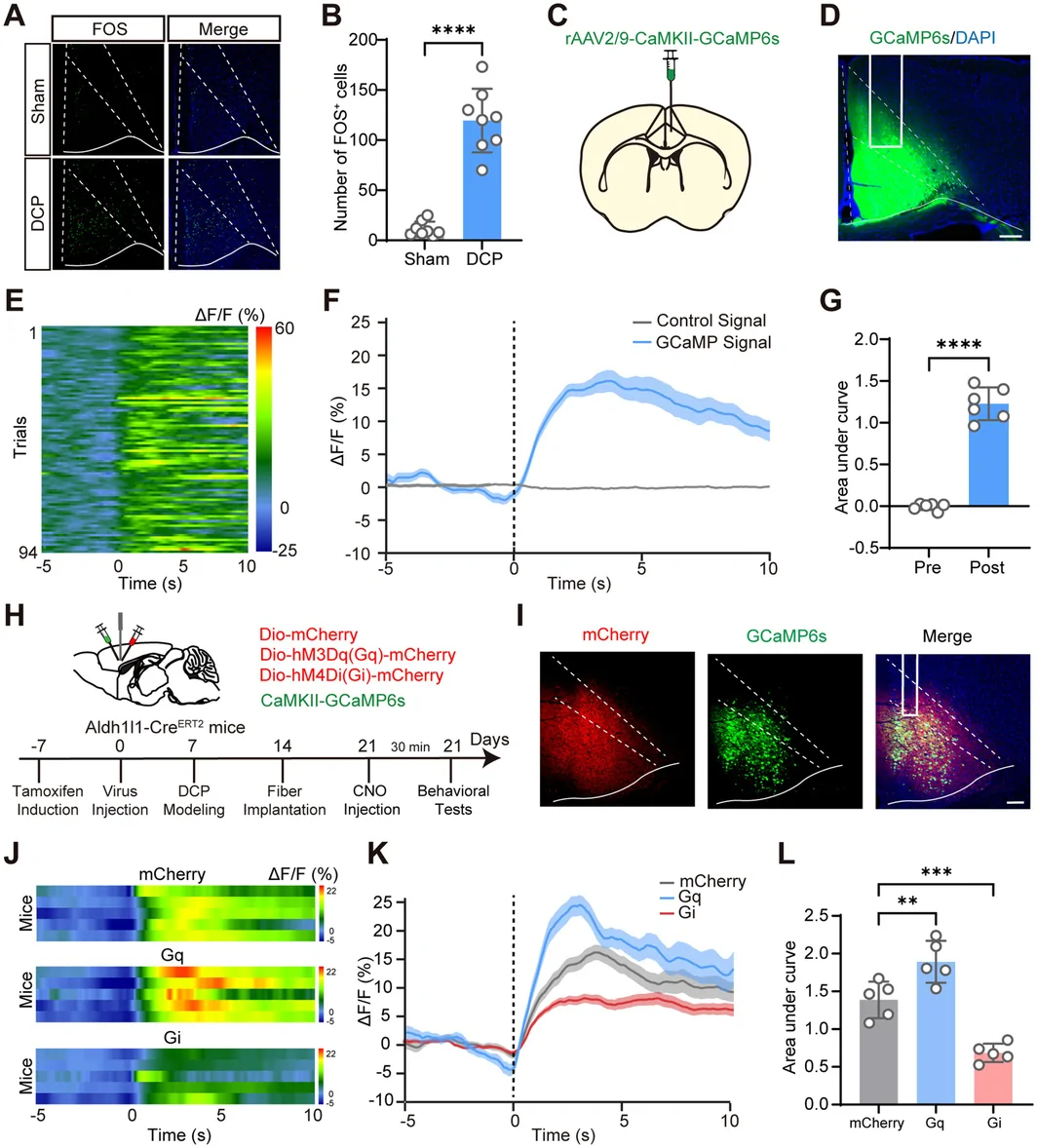
ACC astrocytes positively regulate calcium activity of CaMKII^+^ neurons. (A) Representative images of FOS staining in the ACC. Scale bar = 200 μm. (B) Quantification of FOS‐positive neurons in response to DCP‐induced scratching behaviors (*n* = 8 per group). (C) Schematic illustration of viral delivery for fiber photometry recording of calcium activity in ACC CaMKII^+^ excitatory neurons. (D) Histological verification of viral expression and optical fiber implantation in the ACC. Scale bar = 200 μm. (E) Heatmap of GCaMP6s fluorescence aligned to scratching bout onset across all mice. (F) Mean fluorescent response to scratching behavior. (G) Area under the curve showing fluorescence changes during pre‐ and post‐scratching periods (*n* = 6 per group). (H) Schematic illustration of chemogenetic manipulation of astrocytes and calcium signal recording of CaMKII^+^ neurons in the ACC of Aldh1l1‐Cre^ERT2^ mice. (I) Representative images of mCherry and GCaMP6s fluorescence. Scale bar = 100 μm. (J) Heatmap of GCaMP6s fluorescence aligned to scratching bout onset across all mice. (K) Mean fluorescent response to scratching behavior. (L) Area under the curve showing fluorescence changes during pre‐ and post‐scratching periods. *n* = 5 per group. Data are presented as mean ± SD, **p < 0.01, ***p < 0.001, ****p < 0.0001.

To identify the involvement of ACC neurons in DCP‐induced chronic itch, FOS immunofluorescence staining was performed. Our data revealed a prominent elevation in FOS^+^ neurons in the ACC of DCP‐treated mice, indicating neuronal activation during chronic itch processing (Figure 5A,B). Given that CaMKII^+^ excitatory neurons constitute the major neuronal subpopulation in the ACC and are critical for mediating sensory and affective signal transduction, we employed fiber photometry to record calcium signals in the ACC CaMKII^+^ neurons (Figure 5C,D). We observed that CaMKII^+^ neurons displayed enhanced calcium signal transients and increased signal AUC during scratching behaviors (Figure 5E–G), highlighting the involvement of ACC CaMKII^+^ excitatory neurons in DCP‐induced chronic itch processing.

We further investigated whether ACC astrocytes functionally modulate the activity of CaMKII^+^ excitatory neurons in DCP‐induced chronic itch [28]. Combing chemogenetic manipulation of ACC astrocytes with calcium signal recording of ACC CaMKII^+^ neurons (Figure 5H,I), we observed that chemogenetic activation of ACC astrocytes significantly enhanced the calcium signal activity of CaMKII^+^ excitatory neurons, while chemogenetic suppression of ACC astrocytes reduced neuronal calcium signals (Figure 5J–L). Collectively, these data demonstrate that ACC astrocytes positively modulate the calcium activity of local CaMKII^+^ excitatory neurons during pruriceptive processing.

### 3.5 DCP Induces Alterations of Gliotransmitter Levels in the ACC

**FIGURE 6:**
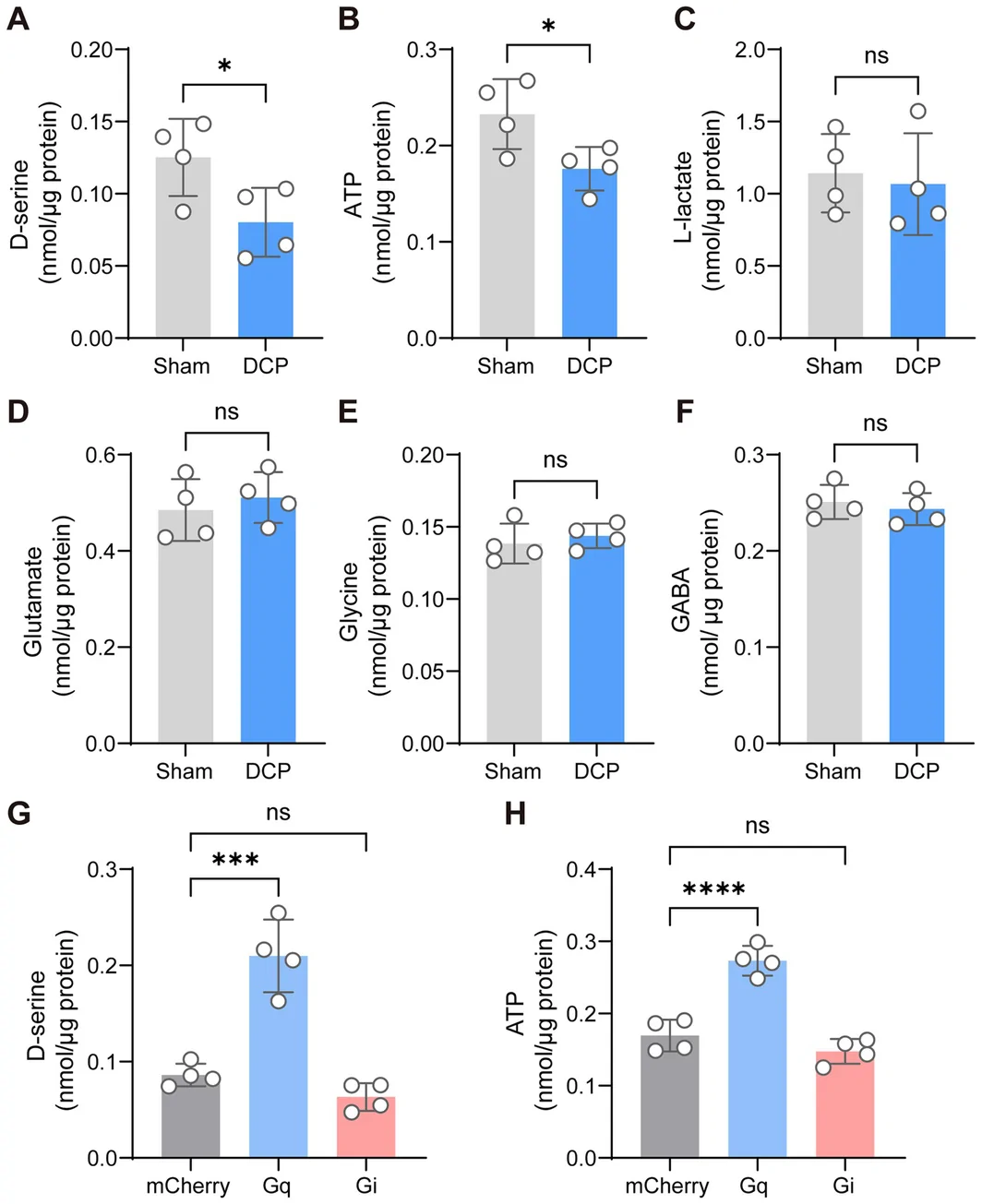
DCP induces decreased levels of D‐serine and ATP in the ACC. Detections of (A) D‐serine, (B) ATP, (C) L‐lactate, (D) glutamate, (E) glycine, (F) GABA in the ACC of Sham and DCP group mice. Detections of (G) D‐serine, (H) ATP in the ACC of mCherry, hM3Dq, and hM4Di group mice. *n* = 4 per group. Data are presented as mean ± SD, *p < 0.05, ***p < 0.001, ****p < 0.0001.

Given that gliotransmitters are key mediators of astrocyte‐neuron communication, we quantified the levels of major gliotransmitters in the ACC. Our results suggested that there was a significant decrease in the levels of D‐serine and ATP in the ACC of DCP‐treated mice (Figure 6A,B). However, no significant changes in the levels of L‐lactate, glutamate, glycine, and GABA were observed (Figure 6C–F). Chemogenetic activation of ACC astrocytes showed a significant increase in the levels of D‐serine and ATP (Figure 6G,H). These results suggest that D‐serine and ATP are key astrocyte‐derived mediators regulating ACC neuron–glia crosstalk in chronic itch and its emotional comorbidities.

## 4 Discussion

In this study, we examined the role of ACC astrocytes in chronic itch processing and comorbid anxiety and depression. We demonstrated that ACC astrocytes are morphologically and functionally activated during chronic itch, and this activation exerts anti‐pruritic, anxiolytic, and anti‐depressant effects. Notably, DCP‐induced chronic itch reduces D‐serine and ATP levels in the ACC, and ACC astrocytes positively modulate the calcium activity of local CaMKII^+^ excitatory neurons and the release of these two gliotransmitters. Our results collectively clarify that ACC astrocytes act as an endogenous cortical modulator of chronic itch and its comorbid affective behaviors, providing novel insights into the cortical glial mechanisms underlying itch perception and emotional processing.

Chronic itch is a globally prevalent and intractable clinical symptom that causes persistent skin damage and severely debilitating comorbidities, including anxiety, depression, and social dysfunction [29, 30, 31]. Pruritus exacerbates psychological distress, which in turn aggravates itch perception, perpetuating a self‐sustaining loop of disease progression [32]. Conventional therapeutic options, including antihistamines, corticosteroids, and immunomodulators, frequently demonstrate limited efficacy and considerable adverse effects, such as skin atrophy, immunosuppression, and somnolence [33]. These therapeutic shortcomings are largely ascribed to an incomprehensive understanding of the central mechanisms. Nevertheless, mounting evidence has increasingly highlighted the contribution of diverse brain regions in itch processing [8, 34]. Functional imaging studies have revealed that itch stimuli activate a distributed neural network encompassing the parabrachial nucleus, periaqueductal gray, insular cortex, hippocampus, amygdala, and ACC [35, 36, 37]. Among these regions, the ACC serves as a core hub integrating the sensory, motivational, and affective dimensions of chronic itch, making it a vital modulator of itch‐emotion comorbidity [11, 38].

Preclinical studies have demonstrated that chemogenetic inhibition of excitatory neurons in the ACC alleviates DCP‐induced scratching behavior, while suppression of the GABAergic neurons exacerbates this response [14]. In our prior work, we observed analogous opposite modulatory roles of ACC pyramidal neurons and GABAergic neurons in acute itch models induced by histamine and chloroquine [9, 36]. Acute itch is a transient, stimulus‐dependent response driven primarily by rapid peripheral‐to‐central signal transmission. In contrast, chronic itch processing is characterized by persistent pathological hypersensitization and circuit remodeling, processes in which astrocytes are key mediators of long‐term neural plasticity, inflammation and structural reorganization [39, 40]. Accumulating evidence confirms that astrocytes are core modulators of neural plasticity and synaptic transmission under physiological and pathological conditions, including chronic pain and emotional disorders [41, 42]. Consistent with findings in the RVM, our results verify that chronic itch induces comprehensive structural remodeling and functional activation of ACC astrocytes [21]. Distinct from uniform neuronal activation, ex vivo calcium imaging revealed compartment‐specific astrocytic plasticity: ATP‐evoked calcium responses were selectively enhanced in the soma and large branches, with no changes in fine terminal territories. This heterogeneous subcellular activation implies functional deficits in astrocytic synaptic terminals during chronic itch, which may disrupt local astrocyte‐neuron signaling.

Gliotransmitter release is the predominant mechanism mediating astrocyte‐neuron crosstalk in neural circuit modulation [43, 44]. Our study found that DCP‐induced chronic itch significantly downregulates ACC D‐serine and ATP levels, which can be fully restored by chemogenetic activation of ACC astrocytes. Functional verification further confirmed that astrocyte activation elevates CaMKII^+^ neuronal activity and alleviates itch and emotional comorbidities, indicating D‐serine and ATP as key functional mediators of ACC glial‐neuronal regulation. Consistent with previous reports, astrocyte‐derived ATP and D‐serine modulate neuronal excitability and emotional behaviors via synaptic receptor regulation [45, 46, 47]. Astrocytes synthesize D‐serine from L‐serine via serine racemase (SR) and release it through calcium‐dependent vesicular exocytosis upon cellular activation. Secreted D‐serine binds to the glycine‐binding site of the NMDA receptor GluN1 subunit, which, together with glutamate binding to the GluN2 subunit, facilitates NMDA receptor channel opening, enhances neuronal calcium influx, and promotes glutamatergic synaptic plasticity [48, 49]. In parallel, activated ACC astrocytes release ATP via calcium‐dependent exocytosis. Extracellular ATP acts on neuronal P2X and P2Y purinergic receptors: P2X activation mediates rapid cation influx to depolarize postsynaptic membranes and increase neuronal firing, whereas P2Y stimulation triggers G‐protein‐coupled signaling to amplify neuronal calcium responses and potentiate presynaptic glutamatergic transmission [50, 51]. Whether the two gliotransmitters function independently or synergistically, and their downstream signaling cascades, remain to be clarified [52]. Further studies are required to explore the synergistic effects of receptor adaptation and tripartite synapse remodeling in astrocyte‐mediated neuronal modulation.

Targeting ACC astrocytes may provide a superior complementary strategy for chronic itch intervention compared with single‐neuron modulation [40]. As astrocytes form extensive functional connections with both excitatory and inhibitory neurons in the ACC, they can globally tune cortical circuit activity and concurrently regulate itch sensation and associated negative emotions [21, 53]. Our chemogenetic experiments validate that specific astrocyte manipulation effectively improves behavioral phenotypes without affecting basic motor function, confirming the safety and specificity of this targeting strategy. Moreover, astrocyte‐derived gliotransmitters represent promising targets for the development of selective therapeutic agents, offering substantial translational potential for clinical application in blocking the vicious itch–distress cycle.

The current study could be further improved from the following aspects. Firstly, all validations were performed in a DCP‐induced allergic chronic itch model. Further verification in multiple itch models and human clinical samples is needed to confirm the universal translational applicability of these findings. Secondly, the ACC is composed of distinct subnuclei, primarily the dorsal and ventral ACC. Emerging evidence has suggested that these two subnuclei exert distinct roles in itch perception and negative emotion regulation [54, 55, 56]. It is worth investigating the distinct roles of astrocytes in different ACC subnuclei, which would help to refine our understanding of the region‐specific glial mechanisms underlying itch‐affective integration.

## 5 Conclusions

In summary, our findings identify the role of ACC astrocytes in modulating chronic itch and its comorbid anxiety and depression. Chronic itch induces morphological and functional remodeling of ACC astrocytes, whose activation exerts anti‐pruritic, anxiolytic, and anti‐depressant effects and enhances the activity of CaMKII^+^ excitatory neurons. Besides, DCP‐induced chronic itch reduces D‐serine and ATP levels in the ACC, and ACC astrocytes positively modulate the release of these two gliotransmitters. These results expand our understanding of the cortical glial mechanisms underlying itch‐affective integration, laying a foundation for the design of new targeted therapies against chronic itch and its associated affective disorders.

## Author Contributions

Jing Huang and Shengxi Wu designed and supervised the study and critically revised the manuscript. Ze Fan, Xiaotong Shi, and You Wu performed the experiments, analyzed the data, and drafted the manuscript. Hui Zhang and Haopeng Zhang contributed to the experimental design. Ningcan Ma, Ziyi Dai, Lisen Wang, Yushan Jin, Yuanyuan Zhu, and Yiming Chen contributed to the experiments, collected, and analyzed the data. All authors read and approved the final manuscript.

## Funding

This work was supported by the Brain Science and Brain‐like Intelligence Technology‐National Science and Technology Major Project (2025ZD0214900), the National Natural Science Foundation of China (82271243, 82221001), Shaanxi Province Key Research and Development Plan (2024SF‐ZDCYL‐01‐13, 2021JCW‐13), and the Ministry of Education Open project of the Key Laboratory of Neurovascular Biology (NV20230017).

## Ethics Statement

This study was approved by the Institutional Animal Care and Use Committee at the Fourth Military Medical University (NO: IACUC‐20250181) and complied with the NIH Guide for the Care and Use of Laboratory Animals.

## Conflicts of Interest

The authors declare no conflicts of interest.

## Supporting information


**Table S1:** Sources of the antibodies used in this study (related to Figures 2 and 5).
**Table S2:** Sources of assay kits used in this study (Figure 6).
**Table S3:** Statistical information for Figures 1–6.

## Data Availability

All data supporting the findings of this study are available from the corresponding author upon reasonable request.
