# Supplementary material for: Anterior Cingulate Cortex Astrocytes Regulate Chronic Itch and Comorbid Negative Emotional Disorders in Mice
Source: CNS Neurosci Ther. 2026 Sep 18;32(9):e71165. doi: 10.1002/cns.71165 (PMC13588149; doi:10.1002/cns.71165)
Supplement: Supplementary file 1 [file CNS-32-e71165-s001.docx]

**Anterior Cingulate Cortex Astrocytes Regulate Chronic Itch and Comorbid Negative Emotional Disorders in Mice**

Ze Fan, Xiaotong Shi, You Wu, Ningcan Ma, Ziyi Dai, Lisen Wang, Yushan Jin, Yuanyuan Zhu, Yiming Chen, Haopeng Zhang, Hui Zhang, Shengxi Wu, Jing Huang

**Supplementary Information**

- Table S1
- Table S2
- Table S3

**Table S1.** Sources of the antibodies used in this study (related to Figure 2 and 5)

| **Target of antibody** | **Source** | **Application** | **Dilution** | **Identifier** |
| --- | --- | --- | --- | --- |
| GFAP | GeneTex | IF | 1:500 | GTX85454 |
| c-fos | Cell Signaling Technology | IF | 1:3000 | 2250 |
| S100β | Abcam | IF | 1:200 | ab227914 |
| Rabbit IgG H&L  (Fluor 488) | Invitrogen | IF | 1:500 | A21206 |
| Chicken IgG H&L  (Fluor 594) | Invitrogen | IF | 1:500 | A11042 |

IF: immunofluorescence.

**Table S2.** Sources of assay kits used in this study (Figure 6)

| **Target of the assay kit** | **Source** | **Country** | **Identifier** |
| --- | --- | --- | --- |
| D-serine | Cosmo Bio | Japan | CSR-CT-DSC-K01 |
| ATP | Abcam | USA | ab83355 |
| L-lactate | Abcam | USA | ab83355 |
| gluatamate | Abcam | USA | ab83389 |
| glycine | Abcam | USA | ab211100 |
| GABA | Cloud-Clone | China | CEA900Ge |

ATP: adenosine triphosphate; GABA: γ-aminobutyric acid.

**Table S3.** Statistical information for Figure 1 to Figure 6

| **Data** | **Response variable** | **Groups** | **Normality test (Shapiro-Wilk)** | **Homogeneity of variance test (Levene’s Test)** | **Hypothesis test** | **Test value** | **P value** |
| --- | --- | --- | --- | --- | --- | --- | --- |
| Fig.1C | Bouts of scratching | Sham (n=10) DCP (n=10) | W=0.8408, p=0.0451 W=0.9410, p=0.5646 | F=1224, p<0.0001 | Mann-Whitney U test | Z=-3.78 | ****p<0.0001 |
| Fig.1D | Total distances (cm) | Sham (n=10) DCP (n=10) | W=0.9126, p=0.2995 W=0.9034, p=0.2384 | F=1.284, p=0.7155 | Two-tailed unpaired t test | t=0.05119, df=18 | p=0.9597 |
|  | Time in center zone (%) | Sham (n=10) DCP (n=10) | W=0.8981, p=0.2088 W=0.9748, p=0.9314 | F=5.842, p=0.0148 | Welch’s t test | t=3.716, df=11.99 | **p=0.0030 |
|  | Entries number | Sham (n=10) DCP (n=10) | W=0.9806, p=0.9684 W=0.9482, p=0.6467 | F=2.778, p=0.1441 | Two-tailed unpaired t test | t=6.261, df=18 | ****p<0.0001 |
| Fig.1E | Total distances (cm) | Sham (n=10) DCP (n=10) | W=0.9735, p=0.9214 W=0.9187, p=0.3463 | F=2.370, p=0.2147 | Two-tailed unpaired t test | t=1.309, df=18 | p=0.2071 |
|  | Time in open arm (%) | Sham (n=10) DCP (n=10) | W=0.9368, p=0.5175 W=0.8834, p=0.1426 | F=1.724, p=0.4295 | Two-tailed unpaired t test | t=3.335, df=18 | **p=0.0037 |
|  | Entries number | Sham (n=10) DCP (n=10) | W=0.9694, p=0.8848 W=0.9110, p=0.2876 | F=1.262, p=0.7345 | Two-tailed unpaired t test | t=3.295, df=18 | **p=0.0040 |
| Fig.1G | Immobility duration in TST (s) | Sham (n=10)  DCP (n=10) | W=0.8169, p=0.0232 W=0.9523, p=0.6962 | F=2.074, p=0.2922 | Mann-Whitney U test | Z=-2.83 | **p=0.0031 |

| **Data** | **Response variable** | **Groups** | **Normality test (Shapiro-Wilk)** | **Homogeneity of variance test (Levene’s Test)** | **Hypothesis test** | **Test value** | **P value** |
| --- | --- | --- | --- | --- | --- | --- | --- |
| Fig.1H | Immobility duration in FST (s) | Sham (n=10) DCP (n=10) | W=0.9758, p=0.9389 W=0.9498, p=0.6664 | F=3.120, p=0.1054 | Two-tailed unpaired t test | t=2.234, df=18 | *p=0.0384 |
| Fig.1I | Sucrose preference (%) | Sham (n=10) DCP (n=10) | W=0.99697, p=0.8885 W=0.9243, p=0.3944 | F=10.48, p=0.0017 | Welch’s t test | t=3.204, df=10.70 | **p=0.0087 |
| Fig.1J | Latency to contact food (s) | Sham (n=10) DCP (n=10) | W=0.9862, p=0.9896 W=0.9500, p=0.6689 | F=1.261, p=0.7354 | Two-tailed unpaired t test | t=0.5205, df=18 | p=0.6091 |
| Fig.1K | Latency to eat food (s) | Sham (n=10) DCP (n=10) | W=0.9471, p=0.6582 W=0.9840, p=0.9828 | F=4.529, p=0.0447 | Welch’s t test | t=2.700, df=13.07 | *p=0.0181 |
| Fig.1L | Food consumption (g) | Sham (n=10) DCP (n=10) | W=0.9370, p=0.5206 W=0.9101, p=0.2816 | F=1.423, p=0.6076 | Two-tailed unpaired t test | t=0.3687, df=18 | p=0.7167 |
| Fig.2B | Number of S100β^+^ cells (per visual field) | Sham (n=3) DCP (n=3) | W=0.9423, p=0.5367 W=0.9231, p=0.4633 | F=1.668, p=0.7442 | Two-tailed unpaired t test | t=0.2115, df=4 | p=0.8429 |
| Fig.2C | Relative fluorescence intensity (%) (GFAP^+^ S100β^+^/S100β^+^) | Sham (n=3) DCP (n=3) | W=0.9393, p=0.5245 W=0.8598, p=0.2670 | F=2.445, p=0.5806 | Two-tailed unpaired t test | t=6.880, df=4 | **p=0.0023 |

| **Data** | **Response variable** | **Groups** | **Normality test (Shapiro-Wilk)** | **Homogeneity of variance test (Levene’s Test)** | **Hypothesis test** | **Test value** | **P value** |
| --- | --- | --- | --- | --- | --- | --- | --- |
| Fig.2F | Astrocyte area (μm^2^) | Sham (n=27) DCP (n=27) | W=0.8567, p=0.0016 W=0.9449, p=0.1607 | F=2.207, p=0.0484 | Mann-Whitney U test | Z=-2.45 | *p=0.0137 |
| Fig.2G | Total process length (μm) | Sham (n=27) DCP (n=27) | W=0.8912, p=0.0085 W=0.9313, p=0.0764 | F=2.534, p=0.0210 | Mann-Whitney U test | Z=-2.22 | *p=0.0256 |
| Fig.2H | Total branch points/cell | Sham (n=27)  DCP (n=27) | W=0.8838, p=0.0058 W=0.9303, p=0.0704 | F=2.251, p=0.0432 | Mann-Whitney U test | Z=-2.29 | *p=0.0212 |
| Fig.2I | Number of intersections | Sham (n=27)  DCP (n=27) | All subgroups meet p>0.05 | All subgroups meet p>0.05 | Two-way ANOVA | F (8, 295) = 3.081 | **P < 0.01 |
| Fig.2O | Area under curve | Sham (n=6)  DCP (n=6) | W=0.9410, p=0.6671 W=0.9017, p=0.3839 | F=454315, p<0.0001 | Welch’s t test | t=5.360, df=5 | **p=0.0030 |
| Fig.3B | Area under curve (somata) | Sham (n=15) DCP (n=21) | W=0.8798, p=0.0471 W=0.9447, p=0.2694 | F=6.622, p=0.0008 | Mann-Whitney U test | Z=-3.83 | ****p<0.0001 |
|  | Peak value  (somata) | Sham (n=15) DCP (n=21) | W=0.8732, p=0.0376 W=0.9089, p=0.0523 | F=9.178, p=0.0001 | Mann-Whitney U test | Z=-3.42 | ***p=0.0004 |

| **Data** | **Response variable** | | **Groups** | | **Normality test (Shapiro-Wilk)** | | **Homogeneity of variance test (Levene’s Test)** | | **Hypothesis test** | | **Test value** | | **P value** |
| --- | --- | --- | --- | --- | --- | --- | --- | --- | --- | --- | --- | --- | --- |
| Fig.3C | Area under curve  (Major branches) | | Sham (n=18) DCP (n=19) | | W=0.7412, p=0.0003 W=0.8452, p=0.0056 | | F=4.353, p=0.0039 | | Mann-Whitney U test | | Z=-3.07 | | **p=0.0016 |
|  | Peak value  (Major branches) | | Sham (n=18) DCP (n=18) | | W=0.7771, p=0.0007 W=0.9280, p=0.1791 | | F=2.689, p=0.0487 | | Mann-Whitney U test | | Z=-3.20 | | ***p=0.0010 |
| Fig.3D | Area under curve (Territories) | | Sham (n=20) DCP (n=20) | | W=0.8713, p=0.0124 W=0.9232, p=0.1143 | | F=1.619, p=0.3023 | | Mann-Whitney U test | | Z=-1.89 | | p=0.0596 |
|  | Peak value  (Torritories) | | Sham (n=18) DCP (n=17) | | W=0.8807, p=0.0268 W=0.8412, p=0.0079 | | F=1.436, p=0.4669 | | Mann-Whitney U test | | Z=-1.39 | | p=0.1729 |
| Fig.4C | Bouts of scratching | | mCherry (n=9) Gq (n=9) Gi (n=9) | | W=0.8264, p=0.0407  W=0.8821, p=0.1651  W=0.9113, p=0.3248 | | F(2, 24)=1.398, p=0.2666 | | Kruskal-Wallis test | | H=15.25 | | ***p=0.0005 |
|  | Post hoc test | | mCherry vs Gq  mCherry vs Gi | |  | |  | | Dunn multiple comparison | | Z=3.787  Z=1.069 | | ***p=0.0003  p=0.5700 |
| Fig.4D | Total distances (cm) | | mCherry (n=10) Gq (n=10) Gi (n=10) | | W=0.8596, p=0.0755  W=0.8622, p=0.0809  W=0.9699, p=0.8903 | | F(2, 27)=1.633, p=0.2140 | | One-way ANOVA | | F(2,27)=2.077 | | p=0.1449 |
| **Data** | **Response variable** | | **Groups** | | **Normality test (Shapiro-Wilk)** | | **Homogeneity of variance test (Levene’s Test)** | | **Hypothesis test** | | **Test value** | | **P value** |
| Fig.4D | Post hoc test | | mCherry vs Gq  mCherry vs Gi | |  | |  | | Dunnett T multiple comparison | | q=0.7698  q=1.249 | | p=0.6628  p=0.3644 |
| Fig.4D | Time in center zone (%) | | mCherry (n=10) Gq (n=10) Gi (n=10) | | W=0.9185, p=0.3448  W=0.9653, p=0.8440  W=0.9329, p=0.4766 | | F(2, 27)=0.5077, p=0.6075 | | One-way ANOVA | | F(2,27)=7.691 | | **p=0.0023 |
|  | Post hoc test | | mCherry vs Gq  mCherry vs Gi | |  | |  | | Dunnett T multiple comparison | | q=3.553  q=0.3770 | | **p=0.0027  p=0.9208 |
| Fig.4D | Entries number | mCherry (n=10) Gq (n=10) Gi (n=10) | | W=0.8727, p=0.1074  W=0.8661, p=0.0899  W=0.7482, p=0.0034 | | F(2, 27)=0.5950, p=0.0072 | | Kruskal-Wallis test | | H=12.91 | | **p=0.0016 | |
|  | Post hoc test | mCherry vs Gq  mCherry vs Gi | |  | |  | | Dunn multiple comparison | | Z=0.2809  Z=2.962 | | p>0.9999  **p=0.061 | |
| Fig.4E | Total distances (cm) | mCherry (n=10) Gq (n=10) Gi (n=10) | | W=0.9145, p=0.3137  W=0.8542, p=0.0652  W=0.8358, p=0.0393 | | F(2, 27)=0.4815, p=0.6230 | | Kruskal-Wallis test | | H=0.4382 | | p=0.8033 | |
|  | Post hoc test | mCherry vs Gq  mCherry vs Gi | |  | |  | | Dunn multiple comparison | | Z=0.5970  Z=2.5462 | | p>0.9999  p>0.9999 | |

| **Data** | **Response variable** | **Groups** | | **Normality test (Shapiro-Wilk)** | | **Homogeneity of variance test (Levene’s Test)** | | **Hypothesis test** | | **Test value** | | **P value** | |
| --- | --- | --- | --- | --- | --- | --- | --- | --- | --- | --- | --- | --- | --- |
| Fig.4E | Time in open arm (%) | mCherry (n=10) Gq (n=10) Gi (n=10) | | W=0.9626, p=0.8151  W=0.8542, p=0.0652  W=0.9404, p=0.5575 | | F(2, 27)=1.674, p=0.2065 | | One-way ANOVA | | F(2,27)=4.959 | | *p=0.0146 | |
|  | Post hoc test | mCherry vs Gq  mCherry vs Gi | |  | |  | | Dunnett T multiple comparison | | q=2.483  q=0.4360 | | *p=0.0361  p=0.8720 | |
| Fig.4E | Entries number | | mCherry (n=10) Gq (n=10) Gi (n=10) | | W=0.9564, p=0.7447  W=0.9578, p=0.7601  W=0.8541, p=0.0649 | | F(2, 27)=0.8117, p=0.4547 | | One-way ANOVA | | F(2,27)=1.711 | | p=0.1997 |
|  | Post hoc test | | mCherry vs Gq  mCherry vs Gi | |  | |  | | Dunnett T multiple comparison | | q=0.6473  q=1.177 | | p=0.7443  p=0.4041 |
| Fig.4F | Immobility duration in TST (s) | | mCherry (n=10) Gq (n=10) Gi (n=10) | | W=0.8693, p=0.0981  W=0.9362, p=0.5115  W=0.9693, p=0.8842 | | F(2, 27)=1.071, p=0.3567 | | One-way ANOVA | | F(2,27)=4.771 | | *p=0.0168 |
|  | Post hoc test | | mCherry vs Gq  mCherry vs Gi | |  | |  | | Dunnett T multiple comparison | | q=2.343  q=0.5718 | | *p=0.0490  p=0.7925 |
| Fig.4G | Immobility duration in FST (s) | | mCherry (n=10) Gq (n=10) Gi (n=10) | | W=0.8698, p=0.0994  W=0.9347, p=0.4953  W=0.9322, p=0.4702 | | F(2, 27)=4.600, p=0.0191 | | Welch’s ANOVA | | F(2,17.77)=4.682 | | *p=0.0233 |

| **Data** | **Response variable** | | **Groups** | **Normality test (Shapiro-Wilk)** | **Homogeneity of variance test (Levene’s Test)** | **Hypothesis test** | **Test value** | **P value** |
| --- | --- | --- | --- | --- | --- | --- | --- | --- |
| Fig.4G | Post hoc test | | mCherry vs Gq  mCherry vs Gi |  |  | Dunnett T multiple comparison | t=3.117  t=1.182 | *p=0.0124  p=0.4362 |
| Fig.4H | Sucrose preference (%) | | mCherry (n=10) Gq (n=10) Gi (n=10) | W=0.7713, p=0.0065  W=0.8783, p=0.1249  W=0.9170, p=0.3323 | F(2, 27)=4.902, p=0.0153 | Kruskal-Wallis test | H=11.66 | **p=0.0029 |
|  | Post hoc test | | mCherry vs Gq  mCherry vs Gi |  |  | Dunn multiple comparison | Z=2.718  Z=0.4318 | *P=0.0131  p>0.9999 |
| Fig.4I | Latency to eat food (s) | | mCherry (n=10) Gq (n=10) Gi (n=10) | W=0.9259, p=0.4086  W=0.9659, p=0.8502  W=0.9492, p=0.6589 | F(2, 27)=2.157, p=0.1352 | One-way ANOVA | F(2,27)=7.336 | **p=0.0029 |
|  | Post hoc test | | mCherry vs Gq  mCherry vs Gi |  |  | Dunnett T multiple comparison | q=3.208  q=0.2092 | **p=0.0065  p=0.9685 |
| Fig.4J | Food consumption (g) | | mCherry (n=10) Gq (n=10) Gi (n=10) | W=0.9299, p=0.4466  W=0.9212, p=0.3668  W=0.9357, p=0.5059 | F(2, 27)=0.8182, p=0.4519 | One-way ANOVA | F(2,27)=0.5307 | p=0.5942 |
|  | Post hoc test | | mCherry vs Gq  mCherry vs Gi |  |  | Dunnett T multiple comparison | q=0.6922  q=0.3146 | P=0.7447  P=0.7554 |
| **Data** | | **Response variable** | **Groups** | **Normality test (Shapiro-Wilk)** | **Homogeneity of variance test (Levene’s Test)** | **Hypothesis test** | **Test value** | **P value** |
| Fig.5B | Number of FOS^+^ cells | | Sham (n=8)  DCP (n=8) | W=0.8592, p=0.1018 W=0.9881, p=0.9917 | F=18.60, p=0.0010 | Welch’s t test | t=9.377, df=7.75 | ****p<0.0001 |
| Fig.5G | Area under curve | | Sham (n=6)  DCP (n=6) | W=0.8189, p=0.0864 W=0.9719, p=0.9050 | F=24.50, p=0.0032 | Welch’s t test | t=15.07, df=5.408 | ****p<0.0001 |
| Fig.5L | Area under curve | | mCherry (n=5) Gq (n=5) Gi (n=5) | W=0.9408, p=0.6719  W=0.9557, p=0.7780  W=0.9937, p=0.9909 | F(2, 12)=0.9249, p=0.4231 | One-way ANOVA | F(2,12)=36.48 | ****p<0.0001 |
|  | Post hoc test | | mCherry vs Gq  mCherry vs Gi |  |  | Dunnett T multiple comparison | q=3.545  q=4.957 | **p=0.0075  ***p=0.0006 |
| Fig.6A | D-serine  (nmol/μg protein) | | Sham (n=4)  DCP (n=4) | W=0.9028, p=0.4450 W=0.8700, p=0.2976 | F=1.258, p=0.8546 | Two-tailed unpaired t test | t=2.510, df=6 | *p=0.0459 |
| Fig.6B | ATP  (nmol/μg protein) | | Sham (n=4)  DCP (n=4) | W=0.9432, p=0.6738 W=0.9267, p=0.5753 | F=2.583, p=0.4563 | Two-tailed unpaired t test | t=2.650, df=6 | *p=0.0380 |
| Fig.6C | | L-lactate  (nmol/μg protein) | Sham (n=4)  DCP (n=4) | W=0.9608, p=0.7838 W=0.8536, p=0.2380 | F=1.684, p=0.6791 | Two-tailed unpaired t test | t=0.3371, df=6 | p=0.7476 |
| Fig.6D | | Glutamate  (nmol/μg protein) | Sham (n=4)  DCP (n=4) | W=0.8991, p=0.4268 W=0.9981, p=0.9959 | F=1.487, p=0.7524 | Two-tailed unpaired t test | t=0.6267, df=6 | p=0.5539 |

| **Data** | **Response variable** | **Groups** | **Normality test (Shapiro-Wilk)** | **Homogeneity of variance test (Levene’s Test)** | **Hypothesis test** | **Test value** | **P value** |
| --- | --- | --- | --- | --- | --- | --- | --- |
| Fig.6E | Glycine  (nmol/μg protein) | Sham (n=4)  DCP (n=4) | W=0.8849, p=0.3600 W=0.9887, p=0.9508 | F=2.611, p=0.4514 | Two-tailed unpaired t test | t=0.6655, df=6 | p=0.5305 |
| Fig.6F | GABA  (nmol/μg protein) | Sham (n=4)  DCP (n=4) | W=0.9500, p=0.9378 W=0.9378, p=0.6407 | F=1.128, p=0.9233 | Two-tailed unpaired t test | t=0.608, df=6 | p=0.5655 |
| Fig.6G | D-serine  (nmol/μg protein) | mCherry (n=4) Gq (n=4) Gi (n=4) | W=0.9329, p=0.6115  W=0.9847, p=0.9290  W=0.8357, p=0.1831 | F(2, 9)=1.652, p=0.2448 | One-way ANOVA | F(2,9)=41.97 | ****p<0.0001 |
|  | Post hoc test | mCherry vs Gq  mCherry vs Gi |  |  | Dunnett T multiple comparison | q=7.186  q=1.328 | ***p=0.0001  p=0.3507 |
| Fig.6H | ATP  (nmol/μg protein) | mCherry (n=4) Gq (n=4) Gi (n=4) | W=0.8173, p=0.1371  W=0.9826, p=0.9172  W=0.9355, p=0.6273 | F(2, 9)=0.4774 p=0.6352 | One-way ANOVA | F(2,9)=44.83 | ****p<0.0001 |
|  | Post hoc test | mCherry vs Gq  mCherry vs Gi |  |  | Dunnett T multiple comparison | q=7.321  q=1.540 | ****p<0.0001  p=0.2619 |
